# Effects and Potential Mechanisms of Pioglitazone on Lipid Metabolism in Obese Diabetic KKAy Mice

**DOI:** 10.1155/2014/538183

**Published:** 2014-03-31

**Authors:** Jun Peng, Yi Huan, Qian Jiang, Su-juan Sun, Chun-ming Jia, Zhu-fang Shen

**Affiliations:** State Key Laboratory of Bioactive Substances and Functions of Natural Medicines, Institute of Materia Medica, Chinese Academy of Medical Sciences and Peking Union Medical College, Beijing 100050, China

## Abstract

This study aimed to analyze the effects and potential mechanisms of pioglitazone on triglyceride and cholesterol metabolism in obese diabetic KKAy mice. Pioglitazone was orally administered to KKAy mice over 30 days. Compared to C57BL/6J mice, KKAy mice developed obvious insulin resistance, hepatic steatosis, and hyperlipidemia. Pioglitazone treatment resulted in deteriorated microvesicular steatosis and elevated hepatic triglyceride levels, though plasma triglyceride and free fatty acid levels were reduced by the treatment, compared to nontreated KKAy mice. Plasma alanine aminotransferase activities were also significantly increased. Additionally, pioglitazone increased plasma concentrations of total cholesterol, HDL-cholesterol, and LDL-cholesterol but decreased hepatic cholesterol. Gene expression profiling revealed that pioglitazone stimulated hepatic peroxisome proliferator-activated receptor gamma hyperactivity, and induced the upregulation of adipocyte-specific and lipogenesis-related genes but downregulated of genes involved in triglyceride lipolysis and fatty acid **β**-oxidation. Pioglitazone also regulated the genes expression of hepatic cholesterol uptake and excretion, such as low density lipoprotein receptor (LDL-R) and scavenger receptor type-BI (SR-BI). These results suggested that pioglitazone could induce excessive hepatic triglyceride accumulation, thus aggravating liver steatosis and lesions in KKAy mice. Furthermore, pioglitazone may suppress the clearance of serum cholesterol from the liver predominantly through inhibition of LDL-R and SR-BI expression, thus increasing the plasma cholesterol.

## 1. Introduction

Insulin resistance, a major abnormality underlying type 2 diabetes mellitus (T2DM) and obesity, is defined as the pathophysiological condition of reduced insulin responsiveness in liver, muscle, and adipose tissue [1]. Its presence in nearly all patients with nonalcoholic fatty liver disease (NAFLD) is now well acknowledged [2, 3]. NAFLD and T2DM frequently coexist as they share the pathogenic abnormalities of excess adiposity and insulin resistance [3]. NAFLD comprises a cluster of liver disorders of which the key feature is hepatic lipid accumulation (steatosis) in the absence of pathologies such as viral hepatitis or alcohol abuse [4]. Insulin resistance plays a key role in the pathogenesis of NAFLD by causing an imbalance between factors that favor hepatic lipid accumulation, such as lipid influx and* de novo* lipogenesis (DNL), and factors that ameliorate lipid accumulation, such as lipid export or oxidation [3]. Thiazolidinedione (TZD) insulin sensitizing agents may reverse the abnormalities by ameliorating insulin resistance, which have been used to prevent the progression of NAFLD in clinical studies [5–7].

TZDs are selective agonists of peroxisome proliferator-activated receptor gamma (PPAR*γ*), a nuclear receptor which is most highly expressed in fat tissues and controls the transcription of genes involved in preadipocyte differentiation and fatty acid transport, synthesis, and storage [8]. Studies have reported that PPAR*γ* is expressed at elevated levels in the livers of obese animals, including KKAy mice [9],* ob/ob* mice [10, 11],* db/db* mice [11], and 5HT-2cR mutant mice [11], as well as in lipoatrophic A-Zip/F1 mice [12, 13], all of which develop severe hepatic steatosis. Liver-specific knockout of PPAR*γ* in either lipoatrophic or obese mice causes decreased hepatic lipid accumulation and reduced expression of a series of genes important to lipid metabolism [12, 14]. Accordingly, deletion of PPAR*γ* in hepatocytes protects mice against high-fat diet induced hepatic steatosis [15]. Conversely, hepatic PPAR*γ* overexpression results in exacerbated steatosis by mechanisms involving activation of lipogenic genes, thereby increasing* de novo* lipogenesis and hepatic triglyceride content [16]. These findings imply a strong relationship between hepatic steatosis and elevated PPAR*γ* expression, which suggest that PPAR*γ* agonist administration could increase lipid accumulation in obese or hepatic PPAR*γ* overexpressing mice [9, 11, 13, 17]. However, in methionine-choline deficient diet-induced models of NAFLD, overexpression of PPAR*γ* or treatment with PPAR*γ* agonists, including rosiglitazone and pioglitazone, has been shown to improve hepatic steatosis and liver lesions [18–21], whereas heterozygous deficiency in PPAR*γ* or administration of PPAR*γ* antagonists led to an increase in steatosis and lesions [19]. Similar outcomes were also found in middle-aged male low-density lipoprotein receptor (LDLR)^−/ −^ mice fed a high-fat diet [20]. Moreover, it has been reported that treatment with pioglitazone or rosiglitazone improves hepatic steatosis in patients with NAFLD, although the primary outcome is not always uniform [5–7, 22]. Consequently, the effects of PPAR*γ* on hepatic steatosis have not been conclusive, as the administration of PPAR*γ* agonists in different animal models induces seemingly opposing effects, and the effects also differ between mice and humans, as well as among clinical studies. Thus, whether TZDs administration indeed improves hepatic steatosis in NAFLD is deserved to be systematically explored before using TZDs to prevent the progression of the disease in patients.

Cholesterol is another important category of lipids. Its homeostasis is usually linked to fatty acid metabolism, and its presence varies widely with the state of insulin resistance in NAFLD and T2DM [23, 24]. Clinical studies have shown that treatment with pioglitazone is associated with elevated levels of plasma cholesterol-contained lipoprotein in T2DM, such as low density lipoprotein cholesterol (LDL-C) and high density lipoprotein cholesterol (HDL-C) [25–27]. However, the precise molecular mechanisms involved in producing these effects remain largely unknown. The liver controls the biosynthesis, uptake, and secretion of triglycerides and cholesterol-related lipoproteins, thereby affecting lipid metabolism in the circulation [23, 24]. Evaluating whether pioglitazone affects the hepatic expression of PPAR*γ* and other factors regulating lipid and lipoprotein metabolism may help us to understand the lipid-related mechanisms of action of this drug.

KKAy mice have been used in the past to study the pathogenesis of NAFLD [28]. This widely used animal model of obesity and T2DM exhibits features that are highly comparable to human T2DM, such as insulin resistance, obesity, hyperglycemia, and dyslipidemia accompanied by apparent hepatic steatosis [29]. This study was designed to determine the lipid and lipoprotein effects of pioglitazone on KKAy mice, thereby reconfirming the hepatic lipid effects of PPAR*γ* agonists on obese mice and outlining the potential mechanisms of this process.

## 2. Materials and Methods 

### 2.1. Reagents and Supplies

Pioglitazone hydrochloride was obtained from Jiangsu Hengrui Medicine Co., Ltd. (China). Rabbit anti-FASN antibody, rabbit anti-Acox1 antibody, rabbit anti-SR-BI antibody, rabbit anti-LDLR antibody, and mouse anti-ABCA1 antibody were all purchased from Abcam, Inc. (USA). Rabbit anti-ABCG5 antibody and rabbit anti-ABCG8 antibody were purchased from Santa Cruz Biotechnology, Inc. (USA).

### 2.2. Animal Treatment and Tissue Sampling

Twelve-week-old female spontaneous diabetic KKAy mice and C57BL/6J mice were purchased from the Experimental Animal Center, Chinese Academy of Medical Science, Beijing. Mice were maintained at constant room temperature (22 ± 3°C) in a 12 h light/dark cycle. The KKAy mice were fed* ad libitum* with a high-calorie diet containing 15% protein, 17.2% fat, and 50% carbohydrate for accelerating the development of diabetes. The C57BL/6J mice were fed with standard commercial diet (Research Diets, Inc., Beijing, China). All animal experiments were carried out in strict accordance with the Standards for Laboratory Animals (GB14925-2001) and the Guideline on the Humane Treatment of Laboratory Animals (MOST 2006a) established by the People's Republic of China, and all animal procedures were approved by Beijing Administration Office of Laboratory Animal (approval number: SCXK-Beijing-2009-0004). All efforts were made to minimize suffering.

KKAy mice were prescreened for diabetic features, and only those meeting the following criteria were included in this study: fasting blood glucose > 180 mg/dL, triglycerides > 200 mg/dL, total cholesterol > 150 mg/dL, initial body weight in the range of 45–55 g, and a decreasing percentage of plasma glucose in the range of ±20% at 40 min after insulin injection (0.4 IU/kg, ITT). Twenty of these diabetic animals were then randomly divided into either the vehicle (0.5% CMC-Na) treated group (Con) or the pioglitazone-treated group (Piog). To the ten Piog group mice, pioglitazone was given by oral gavage every day at a dose of 25 mg/kg for one month. Additionally, ten age- and gender-matched C57BL/6J mice were given vehicle as normal controls (Nor). Body weight and food intake were measured every two days. An oral glucose tolerance test (OGTT) was conducted on day 21, and an insulin tolerance test (ITT) was conducted on day 25. At the end of the study, all of the mice were decapitated. Plasma samples were stored for downstream biochemical analysis. The liver and visceral adipose tissues were immediately removed and weighed. Samples were stored at −80°C for subsequent preparation of total RNA and proteins.

### 2.3. Oral Glucose Tolerance Test (OGTT) and Insulin Tolerance Test (ITT)

Animals were fasted for 6 h (for OGTT) or 4 h (for ITT), after which blood was taken from the tail vein and used for glucose assays. Blood taken at 0 min was used as a baseline for each tolerance test. For the OGTT, blood was taken at 30, 60, and 120 min following glucose (2 g/kg) challenge. For the ITT, blood was taken at 40 and 90 min following subcutaneous injection of insulin (0.4 IU/kg).

### 2.4. Plasma Biochemical Analysis

Fasting plasma glucose (FPG) levels were measured by glucose oxidase methods, and plasma levels of triglycerides (TG) and plasma activities of aspartate aminotransferase (AST) and alanine aminotransferase (ALT) were all determined by enzymatic colorimetric methods using commercial kits (BioSino, Inc., China). Fasting plasma insulin (FINS) and adiponectin were measured by ELISA (American Laboratories Product Co., USA, and R&D Systems, Inc., USA, resp.), following manufacturer's instructions. Free fatty acids (FFAs) were measured by commercial kits (Sekisui Medical, Tokyo, Japan). The Homeostasis Model of Assessment-Insulin Resistance (HOMA-IR) was calculated using the following formula: HOMA-IR = (FPG (mg/dL) × FINS (ng/mL))/22.5. Total plasma cholesterol (TC) and HDL-cholesterol (HDL-C) were measured by commercial kits (BioSino, Inc., China). HDL-C was measured in polyethylene glycol-treated plasma. Briefly, ApoB-containing particles were precipitated from plasma by adding 20 *μ*L plasma to 8 *μ*L 20% polyethylene glycol (PEG; P-2139 in 200 mM glycine, pH 10; Sigma-Aldrich) solution. This mixture was incubated at room temperature for 15 min then centrifuged at 1,900 g for 20 min. The supernatant, containing HDL fractions, was removed and used to measure HDL-C concentrations [30]. Plasma concentrations of LDL-cholesterol (LDL-C) were calculated using the following formula: LDL-C = TC − HDL-C − (TG/5) [31].

### 2.5. Liver Histopathology and Lipid Determination

Following treatment, the gallbladder bile from each mouse was collected, and the livers were excised and fixed in 4% paraformaldehyde then paraffin embedded and sectioned at a thickness of 2 *μ*m. Tissue sections were routinely stained with hematoxylin and eosin (H&E) and examined under an optical microscope (Olympus CX41RF, Olympus, Tokyo, Japan). For determination of liver lipids, a 50 mg aliquot of liver was homogenized then the lipids were extracted. The levels of TG, TC, and FFA in liver tissues were determined using a plasma lipids determination kit (BioSino, Inc., China, and Sekisui Medical, Tokyo, Japan). TC concentration in the gallbladder bile was also directly examined using a plasma TC kit (BioSino, Inc., China).

### 2.6. Fecal Total Cholesterol Determination

Mice were housed individually, and the feces were gathered up over the final three days of this study and separated from the bedding. Fecal samples were lyophilized and weighed then grounded up prior to analysis. Dry fecal powder (50 mg) was extracted with 4 mL of methanol: chloroform (1 : 2, v/v) at 37°C for 4 h. One milliliter of supernatant was then removed, evaporated to dryness, and dissolved in 300 *μ*L isopropanol solution with 10% Triton X-100 (v/v). Cholesterol content was determined from 20 *μ*L of this solution, and a commercial kit (BioSino, Inc., China) was used.

### 2.7. Quantitative Real-Time PCR

Total RNA was extracted from frozen liver or adipose tissue using TRIzol reagent (Invitrogen, USA) and further purified. Purified total RNA concentrations and 260/280 nm or 260/230 nm ratios were determined using a Biodropsis BD-2000 spectrophotometer (OSTD Beijing Co., Ltd., China). Total RNA integrity was checked by electrophoresis on agarose gel. cDNA was synthesized using VigoScript First Strand cDNA Synthesis Kit (Vigorous Biotechnology Beijing Co., Ltd., China). For quantitative real-time PCR, cDNA was amplified using 7900 Real-Time PCR System (Applied Biosystems, USA) with SYBR Green Master Mix (Takara, China). The specific primers (see Table S1 in Supplementary Material available online at http://dx.doi.org/10.1155/2014/538183) were designed from sequences available in GeneBank and synthesized by Invitrogen (Beijing, China). Quantitative real-time PCR was carried out using the following protocol: 1 cycle at 95°C for 30 s followed by 40 cycles at 95°C for 5 s and 60°C for 31 s. Results were expressed as fold expression relative to expression in the Con group using the delta-delta Ct (^ΔΔ^Ct) method. The level of *β*-actin mRNA was used as an internal standard.

### 2.8. Western Blot Analysis

Total protein was extracted from frozen liver using RIPA lysis buffer supplemented with a protease and phosphatase inhibitor cocktail (Applygen Technologies Inc., China) and quantitated using a BCA assay. Protein was denatured and prepared for western blot. Equivalent protein amounts were resolved electrophoretically on a 10% sodium dodecyl sulfate polyacrylamide gel and transferred to polyvinylidene difluoride membranes. Membranes were blocked with 5% nonfat milk in Tris-buffered saline (0.1% Tween-20) for 1 h. Blots were incubated overnight at 4°C with primary antibody, followed by incubation with a horseradish peroxidase-conjugated secondary antibody (ZSGB-BIO, Inc., China). The signal was visualized using an enhanced chemiluminescence detection system (ChemiScope 2850, CLiNX Science Instruments, China). Protein band densities were analyzed using the Gel-Pro-Analyzer 3.1 software. The expression of *β*-actin protein was used as an internal standard. Results were expressed as fold expression relative to expression in the Con group.

### 2.9. Statistical Analysis

All values are presented as mean ± S.E.M. Statistical analyses were performed between C57BL/6J mice and vehicle-treated KKAy mice (diabetic effect) and between vehicle-treated KKAy mice and pioglitazone-treated mice (treatment effect). Differences were determined using one-way ANOVA with post hoc tests to compare to vehicle-treated KKAy mice group. Outcomes of *P* < 0.05 were considered to be statistically significant.

## 3. Results

### 3.1. Pioglitazone Ameliorated Systemic Insulin Resistance in KKAy Mice

**Figure 1:**
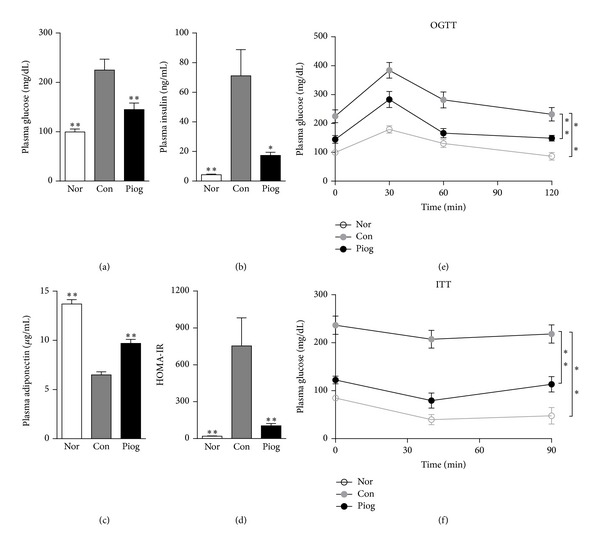
Pioglitazone ameliorated systemic insulin resistance in diabetic KKAy mice. Fasting plasma glucose (a), insulin (b), and adiponectin (c) were measured at the end of the treatment. HOMA-IR (d) was calculated by (FPG (mg/dL) × FINS (ng/mL))/22.5. OGTT (e) and ITT (f) were performed in fasted animals on days 21 and 25 of treatment, respectively, as described in Section 2. Nor, C57BL/6J mice; Con, vehicle-treated KKAy mice; Piog, pioglitazone-treated KKAy mice. Data are presented as mean ± S.E.M. (*n* = 10 per group). *P < 0.05, **P < 0.01 versus Con.

As shown in Figure 1, diabetic KKAy mice exhibited much higher plasma glucose and insulin levels and lower plasma adiponectin concentrations than C57BL/6J mice. Moreover, results of OGTT and ITT showed extreme glucose intolerance and insulin insensitivity in KKAy mice, demonstrating the significant systemic insulin resistance of diabetic KKAy mice (Figure 1(d)). After 30 days of treatment with pioglitazone, the higher plasma glucose and insulin levels as well as lower plasma adiponectin levels in KKAy mice were markedly reversed. Furthermore, pioglitazone-treated mice displayed obvious improvements in glucose tolerance and insulin sensitivity as measured by OGTT and ITT (Figures 1(e) and 1(f)), which was confirmed by a decrease of HOMA-IR (Figure 1(d)). As expected, pioglitazone ameliorated the systemic insulin resistance of diabetic KKAy mice.

### 3.2. Pioglitazone Reduced Plasma TG and FFA Levels but Exacerbated Hepatic Steatosis and Liver Lesions in KKAy Mice

**Figure 2:**
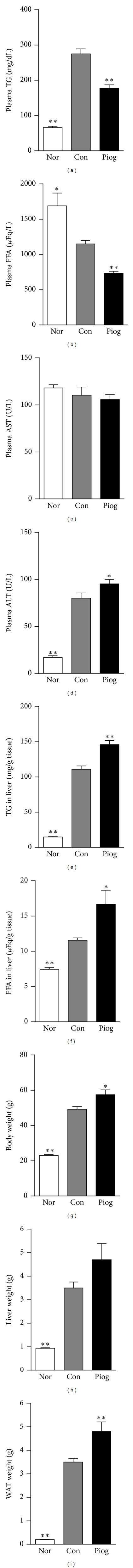
Effects of pioglitazone on biochemical variables involved in fatty acid metabolism and tissue weight in KKAy mice. The levels of plasma TG (a), plasma FFA (b), plasma levels of AST (c), plasma levels of ALT (d), liver TG (e), and FFA (f) were measured by commercial kits. The final body weight (g), liver weight (h) and WAT weight (i) were recorded at the end of the treatment. Nor, C57BL/6J mice; Con, vehicle-treated KKAy mice; Piog, pioglitazone-treated KKAy mice. Data are presented as mean ± S.E.M. (*n* = 10 per group). *P < 0.05, **P < 0.01 versus Con.

**Figure 3:**
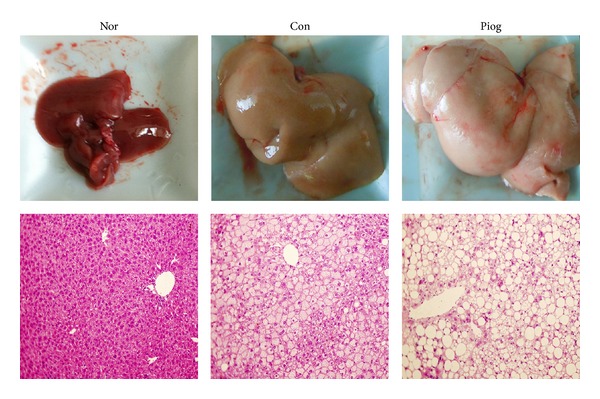
Pioglitazone-induced effects on liver histology of KKAy mice. Representative liver photos and photomicrographs (200×) of H&E-stained liver sections from C57BL/6J mice (Nor), vehicle-treated KKAy mice (Con), and pioglitazone-treated KKAy mice (Piog).

Obese diabetic KKAy mice developed marked hepatic steatosis, which was visualized by proportional increases in the size and coloration (more amber in color) of their livers (Figure 3). The body weight, liver weight, and visceral white adipose tissues (WAT) weight were increased compared to C57BL/6J mice (Figures 2(g), 2(h), and 2(i)). Biochemical and histological analysis of hepatic lipid contents and distribution also revealed that the quantity of lipids was significantly increased in these obese mice (Figures 2(e), and 2(f), and Figure 3). KKAy mice also exhibited much higher plasma levels of TG, FFA, and ALT than C57BL/6J mice (Figures 2(a), 2(b), and 2(d)). Following the 30-day treatment with pioglitazone, the higher plasma levels of TG and FFA in KKAy mice were expectedly reversed; however, unexpectedly, liver steatosis was markedly aggravated. This effect was confirmed by H&E stain of tissue samples (Figure 3), showing a small percentage of microvesicular fat droplets and large areas of macrovesicular hepatocellular vacuolation. The liver weight in pioglitazone-treated KKAy mice was increased by 34% (*P* = 0.075, Figure 2(h)), and the liver became more pale in color (Figure 3). The final body weight and WAT weight were increased by 17% (*P* < 0.05) and 37% (*P* < 0.01), respectively. Furthermore, the treatment increased liver TG content from 110.9 ± 4.7 to 145.9 ± 5.9 mg/g liver (*P* < 0.01); FFA concentrations in the liver were also significantly elevated. Therefore, these data indicated that pioglitazone treatment could stimulate the accumulation of fat droplets in obese KKAy mice, thereby exacerbating the hepatic steatosis. In addition, plasma AST levels (Figure 2(c)) were not changed in KKAy mice by pioglitazone treatment, but plasma ALT levels were slightly increased from 80.1 ± 5.4 to 95.3 ± 4.6 U/L (Figure 2(d), *P* < 0.05), which further confirmed the liver lesion due to the augmented hepatic steatosis.

### 3.3. Pioglitazone Upregulated the Expression of PPAR*γ* Target Genes in the Liver of KKAy Mice

**Figure 4:**
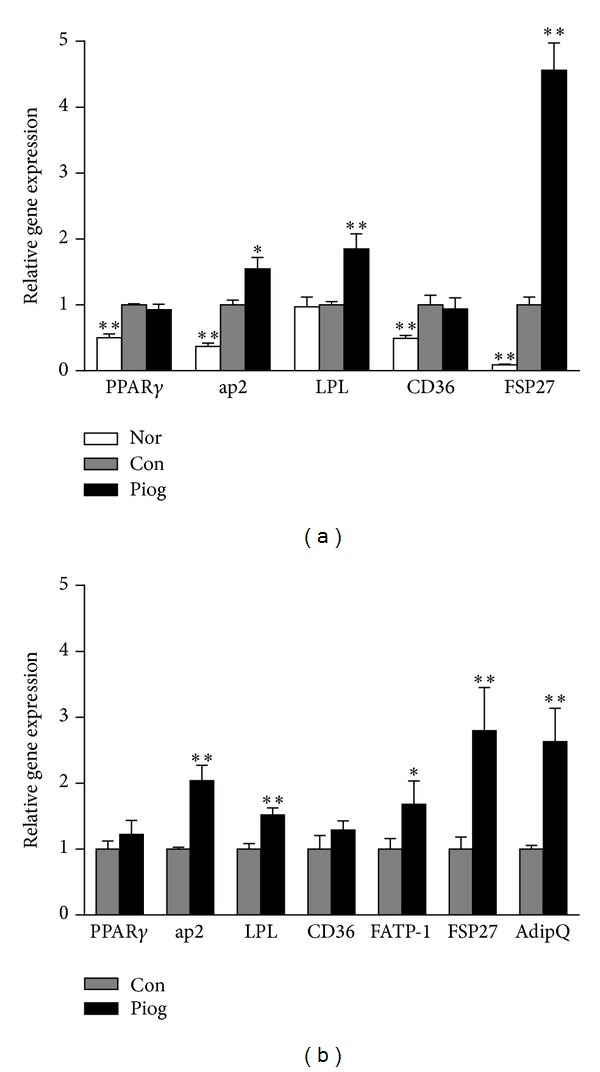
Expression of adipocyte-specific genes in the liver and WAT of vehicle- and pioglitazone-treated KKAy mice. Gene expression in liver (a) and WAT (b), which was examined by using the real-time PCR. Results were expressed as fold change, after correction for *β*-actin levels, relative to the control mice. Nor, C57BL/6J mice; Con, vehicle-treated KKAy mice; Piog, pioglitazone-treated KKAy mice. Data are presented as mean ± S.E.M. (*n* = 6–8 per group). *P < 0.05, **P < 0.01 versus Con. PPAR*γ*, peroxisome proliferator activated receptor gamma; FABP4/ap2, adipocyte fatty acid binding protein 4; LPL, lipoprotein lipase; FAT/CD36, fatty acid translocase/CD36 antigen; FSP27, fat specific protein 27; AdipoQ, adiponectin.

Liver mRNA levels of PPAR*γ* and several PPAR***γ***target genes, including FABP4/ap2, FAT/CD36, LPL, and FSP27, were examined. As expected, KKAy mice had markedly increased expression of PPAR*γ* and its target genes ap2, CD36, and FSP27 in the liver compared to C57BL/6J mice (Figure 4(a)). Following treatment with pioglitazone, aP2, LPL, and FSP27 mRNA levels in the liver of KKAy mice were further increased. In particular, FSP27 was sharply upregulated (4.5-fold) by pioglitazone. These changes suggested that elevated expression of these adipocyte-specific genes may increase the incorporation of fatty acids from the blood into the liver, and fat deposition could be accelerated by pioglitazone. Likewise, the adiponectin gene AdipoQ and adipogenesis-associated genes ap2, CD36, LPL, FATP-1, and FSP27 were upregulated in the WAT.

### 3.4. Effects of Pioglitazone on the Gene Expression of Other Factors Involved in Hepatic Steatosis in the Liver of KKAy Mice

Since several factors may promote the development of hepatic steatosis in KKAy mice, the effects of pioglitazone treatment on other factors regulating lipid and lipoprotein homeostasis in the livers of KKAy mice were examined. As shown in Table 1, a series of genes involved in* de novo *lipogenesis, steatolysis, fatty acid *β*-oxidation, lipoprotein assembling, and metabolism were notably altered in the liver of KKAy mice relative to C57BL/6J mice. Notably, although SREBP expression was decreased, pioglitazone treatment induced the expression of FASN (2.5-fold), SCD-1 (2.11-fold), and DGAT-2 (1.74-fold) mRNA, implying that* de novo *lipogenesis in the liver of KKAy mice was augmented. Secondly, CPT-1a, the rate-limiting enzyme in fatty acid *β*-oxidation, and HSL were strikingly inhibited by pioglitazone treatment, although other genes related to lipolysis and *β*-oxidation either stayed the same (ATGL, FATP-1, mCAD) or increased (Acox1). Finally, ApoB, MTTP, ApoC2, and ApoC3 were significantly downregulated by pioglitazone. To assess the effects of treatment on translation, the protein expressions of two genes, FASN and Acox1, were determined. These genes were selected because they exhibited large changes following treatment and mediate opposing effects in fatty acid metabolism. Consistent with the mRNA levels change, the expressions of FASN (*P* < 0.01) and Acox1 (*P* < 0.05) were both markedly increased by pioglitazone treatment (Figures S1(A) and S1(B)).

### 3.5. Effects of Pioglitazone on Cholesterol Metabolism and Associated Gene Expression in the Liver of KKAy Mice

**Figure 5:**
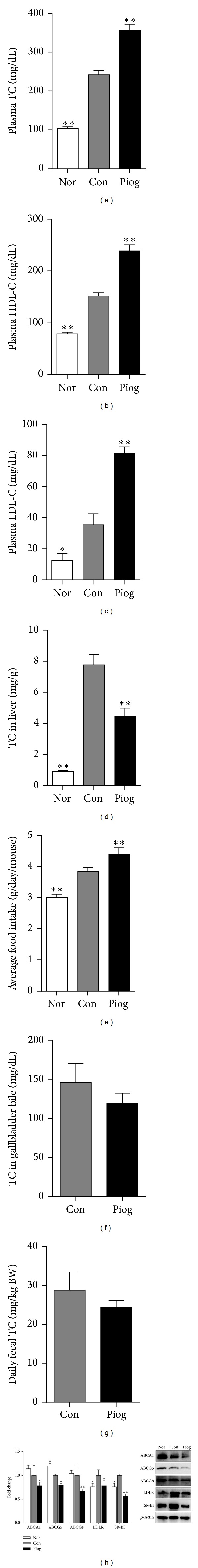
Effects of pioglitazone on cholesterol metabolism and associated hepatic protein expression in KKAy mice. The plasma TC (a), plasma HDL-C (a), plasma LDL-C (c), the levels of liver TC (d), the average food intake (e), gallbladder bile TC (f), and fecal TC (g) were measured as mentioned in Section 2. (h) Representative western blot for ABCA1, ABCG5, ABCG8, LDLR, SR-BI, and *β*-actin is shown. The lower left bar graph represents statistical data from six individual mice per group. The bands were determined by densitometric analysis and expressed as fold change, after correction for *β*-actin levels, relative to the control mice. Nor, C57BL/6J mice; Con, vehicle-treated KKAy mice; Piog, pioglitazone-treated KKAy mice. Data are presented as mean ± S.E.M. (*n* = 10 for biochemical analysis and *n* = 6 for protein analysis). *P < 0.05, **P < 0.01 versus Con.

As shown in Figure 5, KKAy mice had higher concentrations of plasma TC, HDL-C, and LDL-C than C57BL/6J mice, while pioglitazone treatment further increased those levels by 50%, 57%, and 130% (Figures 5(a), 5(b), and 5(c)), respectively. KKAy mice developed hepatic steatosis which was accompanied by increased liver total cholesterol levels. Pioglitazone, however, induced a significant drop of TC in the liver (Figure 5(d)). Moreover, there was a trend toward reduced TC levels in the gallbladder bile in pioglitazone-treated mice, as well as the fecal TC though pioglitazone induced polyphagia (Figure 5(e)) may increase food cholesterol intake. To clarify the potential mechanisms of these effects of pioglitazone on cholesterol metabolism, we evaluated the expression of genes and proteins involved in cholesterol synthesis, uptake, and excretion in the liver of KKAy mice (Table 2 and Figure 5(h)). Of note, pioglitazone-treated mice showed significantly lower expression of SREBP-2, HMGCR, LXR*α*, and LXR*β*, with a slight decrease of ApoAI and FXR mRNA. In line with these findings, genes involved in cholesterol hepatic uptake (SR-BI, ApoE), excretion (ABCBG5, ABCG8), and bile acid synthesis (Cyp7a1, Cyp27a1) and excretion (ABCB11) were all downregulated by pioglitazone. Protein expression of these genes was similarly decreased (Figure 5(h)). The protein expression levels of ABCA1, ABCG5, ABCG8, and SR-BI were all significantly reduced in the liver after pioglitazone treatment. The protein expression of LDL-R was also decreased, although its mRNA levels remained unchanged.

## 4. Discussion

In the present study, we confirmed that KKAy mice developed obvious hepatic steatosis, manifesting in massive hepatic lipid accumulation and accompanied with upregulation of the PPAR*γ* gene as well as adipocyte-specific gene in the liver. Moreover, administration of the PPAR*γ* agonist, pioglitazone, for 30 days exacerbated the development of fatty liver and increased liver lesions. The expression of genes involved in adipogenesis or lipogenesis was further upregulated by pioglitazone. Hepatic TG accumulation may be due to an increase in dietary uptake of FFA, leading to incorporation in the liver, by an increase in hepatic* de novo* FFA synthesis, by a decrease in *β*-oxidation of FFA, and/or by a decrease in secretion of lipoproteins into blood. It is well recognized that PPAR***γ*** and its target genes, such as ap2, CD36, LPL,and FSP27, are predominantly expressed in adipose tissue and have either no detectable or very low basal expression levels in the liver of normal animals [8, 32]. They are involved in fatty acid transportation and fat droplet deposition. In fact, in pioglitazone-treated KKAy mice, the expression of adipocyte marker genes, ap2 and LPL, were significantly elevated not only in WAT but also in the liver (Figure 4). These results suggest that pioglitazone induced PPAR*γ* hyperactivity in the liver of KKAy mice. Elevated expression of the adipocyte-specific genes stimulates not only incorporation of FFA from the blood into WAT but also into the liver, and that fat deposition could be accelerated. Additionally, the hepatic expression of FSP27, a recently identified direct mediator of PPAR*γ*-dependent hepatic steatosis [32], was sharply increased by pioglitazone. Thus, PPAR*γ* overexpression in KKAy mice and pioglitazone-mediated PPAR*γ* hyperactivity may lead to adipogenic hepatic steatosis or hepatic adiposis.

Furthermore, hepatic gene expression of several key factors involved in* de novo* lipogenesis was also markedly upregulated in KKAy mice after pioglitazone treatment. Although the lipogenic genes FASN, SCD-1, and ACC are not target genes of PPAR*γ*, previous studies have shown that overexpression of hepatocyte PPAR*γ* and treatment with PPAR*γ* agonists can promote expression of these genes [11, 16], whereas ablation of PPAR*γ* in the liver decreases their expression and eliminates the response to PPAR*γ* agonists in obese mice [12, 14, 15]. Consistent with these observations, we also showed that pioglitazone enhanced lipogenic gene expression in KKAy mice. Moreover, western blot analysis demonstrated a 1.7-fold (*P* < 0.01) increase in FASN protein and a 1.4-fold (*P* < 0.01) increase in total ACC (Figures S1(A) and S1(C)), indicating that changes in protein levels paralleled changes in the corresponding mRNA levels. However, an important transcription factor, SREBP-1, which closely relates to lipid metabolism and promotes the expression of multiple lipogenic genes, including FAS, ACC, and SCD1 [33], was slightly but significantly downregulated by pioglitazone administration in our study. A possible explanation may be that the SREBP-1 processing was promoted by pioglitazone; thereby the expression of the downstream lipogenic genes was still upregulated, although it needs to be further confirmed.

Our results demonstrated that pioglitazone elevated the mRNA and protein expression levels of Acox-1 but reduced mRNA levels of CPT-1a, apoC3, PPAR*α*, and PPAR*β* (Table 1) in the livers of KKAy mice. It is important to note that CPT-1a is a rate-limiting enzyme of FFA *β*-oxidation which is responsible for FFA import into the mitochondria, whereas Acox-1 is involved in both peroxisomal oxidation and *β*-oxidation of FFAs. ApoC3 is a potent inhibitor of lipoprotein lipase. All of these genes contain PPAR response elements and are activated by PPAR*α* [34]. Consistent with our findings, other PPAR*γ* agonists also upregulate Acox1 gene expression but result in opposing effects on CPT-1 in obese mice [11, 35]. A possible explanation may be that these genes require different coregulators for activation or inhibition by PPAR*γ* agonists, or that they may be under the dominant regulatory control of other transcription factors, such as PPAR*α* and PPAR*β*. CPT-1a may therefore be decreased in a secondary manner via downregulation of PPAR*α* or PPAR*β*. Pioglitazone may therefore impair mitochondrial *β*-oxidation due to reduced import of FFA into the mitochondria, despite elevated Acox1 protein expression levels.

As mentioned above, pioglitazone simultaneously promoted hepatic uptake and* de novo* synthesis of FFA while reducing FFA *β*-oxidation, thus significantly increasing FFA levels in the liver (Figure 2(f)). Additionally, pioglitazone induced a 1.74-fold increase in the expression of DGAT-2, a major enzyme that mediates the incorporation of FFA and glycerol into TG in the liver [36]. Pioglitazone treatment also downregulated gene expression of the lipolytic enzyme HSL, thus possibly reducing lipid degeneration. Furthermore, pioglitazone dramatically decreased the expression of apoB and microsomal triglyceride transfer protein (MTTP) [37]. As the transfer of TGs to lipoproteins (VLDL) involves the cotranslational addition of lipids to apoB in a process catalyzed by MTTP, it is possible that pioglitazone inhibits the assembly of VLDL and thereby reduces the secretion of TGs into blood. Collectively, we conclude that all of these factors contribute to pioglitazone's induction of TG accumulation and the increase in microvesicular lipid droplets in the liver of KKAy mice.

Pioglitazone acts as an insulin sensitizer, decreasing circulating TG and FFA and ameliorating systemic insulin resistance in KKAy mice (Figure 1). The significant decreases in plasma TG and FFA levels were balanced by a substantial TG accumulation within the liver. However, it has been reported that treatment with PPAR*γ* agonists, including pioglitazone, improved hepatic steatosis in patients with NAFLD [5–7, 22], as well as in other animal models of NAFLD [18–21]. Thus, an important unanswered question concerns the similarity between mouse and human hepatic steatosis. We are unaware of any published studies quantitating PPAR*γ* levels in steatotic liver from humans. The levels of PPAR*γ* in liver may be a key factor that determines whether a PPAR*γ* agonist could induce PPAR*γ* hyperactivity. Meanwhile, the different drug dosages used in animal experiments and clinical studies, as well as the hepatic distribution of drug, could also influence the activation degree of PPAR*γ* in the liver. Finally, it is possible that worsening of hepatic steatosis with pioglitazone treatment in obese KKAy mice is a species-specific manifestation, similar to the hepatomegaly caused by PPAR*α* agonists in rodents [38].

In addition to investigating TG and FFA metabolism, the present study also probed the effects of pioglitazone on circulating or hepatic cholesterol metabolism. Clinical studies have demonstrated that pioglitazone treatment increases plasma HDL-C and LDL-C levels but improves LDL particle concentration and size [25–27]. Rahimian et al. [10] reported that there are strong positive correlations between plasma TC or HDL-C and PPAR*γ* levels in the liver of* ob/ob* mice. Consistent with our results, high hepatic expression of PPAR*γ* in KKAy mice has been shown to lead to a marked increase of plasma cholesterol levels relative to C57BL/6j mice. Pioglitazone-induced hyperactivity of PPAR*γ* further elevated the levels of plasma TC, HDL-C, and LDL-C, while the hepatic TC was noticeably decreased (Figure 5(d)). Hepatic cellular cholesterol levels are also controlled by coordinated regulation of biosynthesis, uptake, and secretion. We propose that the pioglitazone-induced reciprocity between liver and plasma cholesterol levels is consistent with an inhibition of normal liver-plasma exchange. Consistent with our expectations, gene analysis and protein detection consistently showed that pioglitazone strongly inhibited the expression of SR-BI in the liver. LDL-R proteins were also significantly diminished despite no alteration in the gene expression levels. LDL-R and SR-BI, respectively, mediate the import of blood LDL-C and HDL-C into the liver [39]. Moreover, apoE gene expression was suppressed, which suggests that the apoE-mediated recognition of LDL was inhibited and that this exacerbated the retention of LDL-C in the blood. It is important to note that HL mRNA expression was downregulated 2-fold by pioglitazone (Table 2). HL exerts both triglyceride lipase and phospholipase activities and preferentially hydrolyzes TG and phospholipids from HDL and LDL, and not from VLDL, thereby leading to a remodeling of HDL favorable to cholesterol uptake by hepatocytes. In addition to its lipolytic activity, HL has been shown to exert a ligand-binding function towards HDL which may enhance the interaction of the lipoprotein with SR-BI, thus facilitating cholesterol uptake [40, 41]. In fact, the activity of HL was shown to be reduced by pioglitazone in liver explants [42]. On the basis of these findings, a depressed circulating activity of HL may be elicited by the reduction of HL mRNAs induced by pioglitazone, and this, together with the decrease in SR-BI proteins, may explain the observed elevated plasma HDL-C levels. Because both liver and WAT LPL expression are increased by pioglitazone and apoC3 expression is decreased, the lipolysis of VLDL/IDL may be accelerated, thereby promoting the production of plasma LDL. Thus the current study observed that the increase of LDL-C (130%) was greater than HDL-C (57%). ApoA1 is the main structural and functional component of HDL and the lipidation of ApoA1 by ABCA1 on the plasma membrane of hepatocytes is a rate-limiting step in plasma HDL formation [43]. ABCA1 expression in liver has been shown to be necessary for HDL formation [44]. A report has shown that PPAR*γ* activates ABCA1 gene transcription but reduces the level of ABCA1 protein in HepG2 cells [45]. However, our results demonstrated that pioglitazone-induced a reduction in the levels of both ABCA1 mRNA and protein. Extrapolating these data to humans, the pioglitazone-induced changes in different cholesterol transporters (ABCA1, SR-BI, LDL-R), apolipoproteins (apoA1, apoC2, apoC3), and lipases (LPL, HL) provide a likely mechanism by which pioglitazone regulates the concentration and size of lipoprotein particles in clinical studies.

We also examined some critical factors involved in hepatic cholesterol synthesis (HMGCR) and excretion (ABCG5/8, NPC1-L1, ABCB4), as well as bile acid synthesis (Cyp7a1, Cyp27a1) and excretion (ABCB11) [23, 46]. To our knowledge, there are very few studies that have directly shown the effects of pioglitazone on the expression of these genes/proteins in the liver. After chronic treatment with pioglitazone, the gene expression levels of Cyp7a1, Cyp27a1, and ABCB11 were significantly decreased, which may suggest a decrease in bile acid synthesis and excretion, thus reducing the conversion of hepatic free cholesterol into bile acid. Several studies have proposed that the PPAR*γ* agonist troglitazone induces intrahepatic cholestasis via the inhibition of ABCB11, potentially contributing to hepatotoxicity [47, 48]. Although the current study did not examine the contents of bile acid in hepatocytes and feces, we are convinced that pioglitazone could not lead to intrahepatic cholestasis because of a likely reduction of bile acid synthesis, and that pioglitazone-induced liver injuries are not associated with these effects. Along these lines, we found that pioglitazone reduced the gene and protein expression of ABCG5/8, which may give rise to a slight decrease of cholesterol in the gallbladder bile and feces (Figures 5(f) and 5(g)). All of the factors mentioned above could affect intrahepatic cholesterol levels in conjunction with the relative contribution of these factors to cholesterol exchange between the circulation, liver, and bile. The present study could not differentiate between these possibilities. These genes above are regulated by LXR and FXR [23, 46], both of which may be affected by PPAR*γ* via a crosstalk with each other (Table 2). Exactly how pioglitazone impacts the expression of these factors involved in cholesterol metabolism remains to be explored.

**Figure 6:**
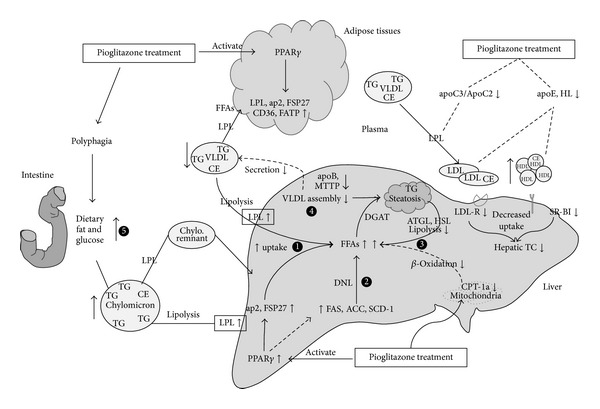
Schematic diagram for the potential mechanisms of pioglitazone exacerbation of hepatic steatosis and increased plasma cholesterol in KKAy mice. Pioglitazone induced hepatic steatosis may be mediated by (1) increased uptake of FFAs from plasma to liver through promoting FFA transporter (LPL, ap2); (2) enhanced* de novo* lipogenesis (DNL) in the liver (FAS, ACC, SCD-1); (3) decreased TGs lipolysis and FFAs *β*-oxidation (HSL, CPT-1a); (4) impaired secretion of lipoprotein (VLDL) into plasma (apoB, MTTP); (5) increased dietary fat absorption. Furthermore, pioglitazone may suppress the clearance of serum cholesterol from the liver predominantly through inhibiting LDL-R and SR-BI expression, thus increasing the plasma levels of cholesterol.

In summary, this study confirmed that chronic treatment with pioglitazone, a PPAR*γ* agonist, not only ameliorates systematic insulin resistance and decreases plasma TG but also exacerbates hepatic steatosis and liver lesions in obese diabetic KKAy mice. As shown in Figure 6, pioglitazone may increase hepatic TG accumulation by a number of mechanisms, including (1) induction of hepatic expression of PPAR*γ* responsive genes that facilitate the incorporation of FFA from the circulation into liver; (2) an enhanced* de novo* lipogenesis; (3) a decreased lipolysis of TG and FFA *β*-oxidation; (4) a reduction in secretion of liver lipids into the circulation; (5) a potential increase in dietary uptake of FFA because of pioglitazone-induced polyphagia (Figure 5(e)). In addition to providing potential mechanisms to account for the effects of pioglitazone on liver FFA and TG metabolism, our results demonstrate that pioglitazone-induced plasma hypercholesterolemia in KKAy mice, or the higher levels of LDL-C/HDL-C observed in diabetic patients treated with pioglitazone, may at least in part be explained by decreased hepatic expression of LDL-R, SR-BI, and HL, resulting in impaired clearance of circulating cholesterol from the liver. It should be noted that the transposition of data from murine to human species remains questionable, and that the reasons why pioglitazone exerts apparently different effects in mouse and human hepatic steatosis, or between different animal models of NAFLD, remain to be elucidated. Meanwhile, T2DM patients with NAFLD should be made aware of the effects of TZDs administration and large, controlled, long-term trials are needed to assess the long-term clinical benefits of pioglitazone for NAFLD patients.

## Supplementary Material

Figure S1: Effects of pioglitazone on the levels of protein FASN, Acox1 and ACC in the liver of KKAy mice.
Table S1: Sequences of primers for real-time PCR.

## Figures and Tables

**Table 1:** Effects of pioglitazone on the expression of fatty acid metabolism related genes in the liver of KKAy mice.

	Nor	Con	Piog
SREBP-1	0.41 ± 0.05**	1.00 ± 0.02	0.81 ± 0.05*
FASN	0.44 ± 0.11**	1.00 ± 0.18	2.50 ± 0.58*
SCD-1	0.98 ± 0.05	1.00 ± 0.16	2.11 ± 0.35**
DGAT-2	1.67 ± 0.09*	1.00 ± 0.27	1.74 ± 0.12**
ACC	0.39 ± 0.06**	1.00 ± 0.07	1.31 ± 0.25
ACOX-1	0.35 ± 0.08**	1.00 ± 0.10	1.80 ± 0.38*
CPT-1a	1.60 ± 0.15**	1.00 ± 0.22	0.56 ± 0.09**
FATP-1	1.90 ± 0.33*	1.00 ± 0.02	0.97 ± 0.10
mCAD	0.38 ± 0.03**	1.00 ± 0.13	1.14 ± 0.07
ApoB	0.35 ± 0.06**	1.00 ± 0.07	0.58 ± 0.06**
ApoC2	0.50 ± 0.04**	1.00 ± 0.07	0.58 ± 0.06**
ApoC3	1.61 ± 0.10**	1.00 ± 0.04	0.49 ± 0.04**
MTTP	1.07 ± 0.23	1.00 ± 0.02	0.60 ± 0.08**
ATGL	2.29 ± 0.19**	1.00 ± 0.04	1.31 ± 0.21
HSL	1.49 ± 0.31	1.00 ± 0.04	0.78 ± 0.03*
PPAR*α*	0.27 ± 0.07**	1.00 ± 0.01	0.81 ± 0.09
PPAR*β*	0.94 ± 0.11	1.00 ± 0.02	0.70 ± 0.04**

The mRNA expression was examined by using the real-time PCR. Results are expressed as fold change, after correction for *β*-actin levels, relative to the control mice. Nor: C57BL/6J mice; Con: vehicle-treated KKAy mice; Piog: pioglitazone-treated KKAy mice. Data are presented as mean ± S.E.M. (*n* = 6–8 per group). *P < 0.05, **P < 0.01 versus Con.

SREBP-1: sterol regulatory element binding transcription factor 1; FASN: fatty acid synthase; SCD1: stearoyl-coenzyme A desaturase 1; DGAT-2: diacylglycerol O-acyltransferase 2; ACC: acetyl-coenzyme A carboxylase; ACOX-1: acyl-coenzyme A oxidase 1; CPT-1a: carnitine-palmitoyltransferase1a; FATP-1: long-chain fatty acid transport protein 1; mCAD: middle-chain acyl-CoA dehydrogenase; ApoB: apolipoprotein B; ApoC2: apolipoprotein C-II; ApoC3: apolipoprotein C-III; MTTP: microsomal triglyceride transfer protein; ATGL: adipose triglyceride lipase; HSL: hormone sensitive lipase; PPAR*α*: peroxisome proliferator activated receptor alpha; PPAR*β*/*δ*: peroxisome proliferator activated receptor delta.

**Table 2:** Effects of pioglitazone on the expression of cholesterol metabolism related genes in the liver of KKAy mice.

	Nor	Con	Piog
SREBP-2	0.50 ± 0.02**	1.00 ± 0.02	0.81 ± 0.03*
HMGCR	1.42 ± 0.23	1.00 ± 0.02	0.75 ± 0.07*
ApoA1	4.41 ± 0.49**	1.00 ± 0.04	0.84 ± 0.11
ApoE	0.75 ± 0.05**	1.00 ± 0.02	0.78 ± 0.05*
SR-BI	0.69 ± 0.05**	1.00 ± 0.02	0.31 ± 0.08**
LDL-R	0.82 ± 0.18	1.00 ± 0.04	1.11 ± 0.09
HL	3.03 ± 0.25**	1.00 ± 0.19	0.48 ± 0.05**
ABCA1	0.14 ± 0.01**	1.00 ± 0.05	0.61 ± 0.03**
ABCG5	1.27 ± 0.10*	1.00 ± 0.06	0.58 ± 0.05**
ABCG8	1.88 ± 0.18**	1.00 ± 0.08	0.55 ± 0.05**
Cyp7a1	1.46 ± 0.19*	1.00 ± 0.07	0.47 ± 0.03**
Cyp27a1	2.44 ± 0.09**	1.00 ± 0.17	0.60 ± 0.04**
ABCB11	2.79 ± 0.15**	1.00 ± 0.06	0.65 ± 0.06**
ABCB4	0.28 ± 0.05**	1.00 ± 0.03	0.98 ± 0.05
NPC1-L1	0.78 ± 0.24	1.00 ± 0.07	0.96 ± 0.22
ACAT-2	1.59 ± 0.17*	1.00 ± 0.04	1.48 ± 0.25
LXR*α*	0.99 ± 0.13	1.00 ± 0.01	0.75 ± 0.10*
LXR*β*	0.93 ± 0.04	1.00 ± 0.03	0.66 ± 0.04**
FXR	0.60 ± 0.09**	1.00 ± 0.05	0.89 ± 0.07

The mRNA expression was examined by using the real-time PCR. Results are expressed as fold change, after correction for *β*-actin levels, relative to the control mice. Nor: C57BL/6J mice; Con: vehicle-treated KKAy mice; Piog: pioglitazone-treated KKAy mice. Data are presented as mean ± S.E.M. (*n* = 6–8 per group). *P < 0.05, **P < 0.01 versus Con.

SREBP-2: sterol regulatory element binding transcription factor 1; HMGCR: 3-hydroxy-3-methylglutaryl-coenzyme A reductase; ApoA1: apolipoprotein A-I; ApoE: apolipoprotein E; SR-BI: scavenger receptor class B, member 1; LDLR: low density lipoprotein receptor; HL: hepatic lipase; ABCA1: ATP-binding cassette, subfamily A, member 1; ABCG5: ATP-binding cassette, subfamily G, member 5; ABCG8: ATP-binding cassette, subfamily G, member 8; Cyp7a1: cytochrome P450, family 7, subfamily a, polypeptide 1; Cyp27a1: cytochrome P450, family 27, subfamily a, polypeptide 1; ABCB11: ATP-binding cassette, subfamily B (MDR/TAP), member 11; ABCB4: ATP-binding cassette, subfamily B (MDR/TAP), member 4; NPC1-L1: Niemann-Pick C1 Like 1; ACAT-2: acetyl-coenzyme A acetyltransferase 2; LXR*α*: liver X receptor alpha; LXR*β*: liver X receptor beta; FXR: farnesoid X receptor.
