# Supplementary material for: Effects and Potential Mechanisms of Pioglitazone on Lipid Metabolism in Obese Diabetic KKAy Mice
Source: PPAR Res. 2014 Mar 31;2014:538183. doi: 10.1155/2014/538183 (PMC3988943; doi:10.1155/2014/538183)
Supplement: Supplementary file 1 — Figure S1: Effects of pioglitazone on the levels of protein FASN, Acox1 and ACC in the liver of KKAy mice. Table S1: Sequences of primers for real-time PCR. [file 538183.f1.zip › mat.538183.v1.pdf]

Supporting information

Table S1. Sequences of primers for real-time PCR.

Gene	5'-Sense primer-3'	5'-Antisense primer-3'	Gene Bank Accession.Version
FABP4/ap2	*TCACCATCCGGTCAGAGAGT*	*TTCATCGAATTCCACGCCCA*	NM_024406.2
LPL	*CGTAGTTCCAGCAGCAAAGC*	*GAAGGCGGTCAAACTCTGGA*	NM_008509.2
FAT/CD36	*CTCCTAGTAGGCGTGGGTCT*	*CACGGGGTCTCAACCATTCA*	NM_001159558.1
FSP27	*ATGGTGCCAGAGTGGTTAGC*	*AGAGTCCCAGGTGAGAGACC*	NM_178373.3
AdipoQ	*CCCAGTCATGCCGAAGATGA*	*CACAAGTTCCCTTGGGTGGA*	NM_009605.4
ACC	*GCTAAACCAGCACTCCCGAT*	*GTATCTGAGCTGACGGAGGC*	NM_133360.2
FASN	*CTTCTGGTGTGCAACTGTGC*	*AGGGCAGCTACCATGTTGTC*	NM_007988.3
SCD1	*GCGCTCTTTACCCTTTGCTG*	*GACTCTCGGGATGGGTGTTC*	NM_009127.4
DGAT-2	*ACTGGAACACGCCCAAGAAA*	*GTAGTCTCGGAAGTAGCGCC*	NM_026384.3
FATP-1	*CGCCGATGTGCTCTATGACT*	*ACACAGTCATCCCAGAAGCG*	NM_011977.3
CPT-1a	*GACCGCGCTCTTAGGACTAC*	*GAGCAGCAAGGAAGGACACT*	NM_013495.2
ACOX-1	*GGAGACAGGTTGTCATCGCT*	*TTCATGACGGAGACCAGTGC*	NM_015729.3
mCAD	*TCAAGATCGCAATGGGTGCT*	*GCTCCACTAGCAGCTTTCCA*	NM_007382.4
ApoB	*GTCCAGGTTGAATCACGGGT*	*AGGATCCTGCAAGGTCAAGC*	NM_009693.2
ApoC2	*GTCTCGGTTCTTCCTGGCTC*	*TCCTTGGCAGAGGTCCAGTA*	NM_009695.3
ApoC3	*GCTAAGTAGCGTGCAGGAGT*	*GCTCCAGTAGCCTTTCAGGG*	NM_023114.3
MTTP	*GTTTTTCCCGGTCAAGCGTT*	*TCTTTCAGTGGGGCGATCTT*	NM_001163457.1
HL	*GTCGCTGCTGGGAACAAAAG*	*GGATCAACTCGCCGATGTCT*	NM_008280.2
ATGL	*TGTGCAAACAGGGCTACAGAG*	*AAAGGGTTGGGTTGGTTCAG*	NM_001163689.1
HSL	*GTGAATGAGATGGCGAGGGT*	*GGAGTCGCGTTAGAGTCACC*	NM_010719.5
SREBP-1	*ACTTTTCCTTAACGTGGGCCT*	*CATCTCGGCCAGTGTCTGTT*	NM_011480.3
SREBP-2	*CATAGCACTGTCCCCGACTC*	*CTCCTTGGCTGCTGACTTGA*	NM_033218.1
PPARγ	*GTCACACTCTGACAGGAGCC*	*AGAACGTGACTTCTCAGCCC*	NM_001127330.1
PPARα	*CCGAACATTGGTGTTCGCAG*	*GATACGCCCAAATGCACCAC*	NM_011144.6
PPARβ	*ATGGGACTCACTCAGAGGCT*	*TGGCTGTTCCATGACTGACC*	NM_011145.3
LXRα	*AGAGTCTTGGGTCGCCAGTA*	*CTGGAGCCCTGGACATTACC*	NM_013839.4
LXRβ	*GCTAGCCATCATCTCGGTCC*	*TCTCAATGGTGGACGCCTTC*	NM_009473.2
FXR	*GGCTGCAAAGGTTTCTTCCG*	*ACATTCAGCCAACATCCCCA*	NM_001163700.1
HMGCR	*TGCCTGGATGGGAAGGAGTA*	*GCCTCGAGTCATCCCATCTG*	NM_008255.2
ApoA1	*GAACGAGTACCACACCAGGG*	*ATGGGCATCAGACTATGGCG*	NM_009692.3
ApoE	*CCTGAACCGCTTCTGGGATT*	*CCATCAGTGCCGTCAGTTCT*	NM_009696.3
SR-BI	*GTGCCCATCATCTGCCAACT*	*TAGGCCTGAATGGCCTCCTT*	NM_016741.2
LDLR	*GGGAACATTTCGGGGTCTGT*	*AGTCTTCTGCTGCAACTCCG*	NM_010700.3

ABCA1	*GCCTGGATCTACTCTGTCGC*	*GCCATTGTCCAGACCCATGA*	NM_013454.3
ABCG5	*CTATCAGCTTGTGGGTGCCA*	*GAGGACGTGTAGCACGTAGG*	NM_031884.1
ABCG8	*AAGACCCTTGGGCGTTACAG*	*CCACTGTGAAAGCCGATCCT*	NM_026180.2
ABCB11	*TCATCGCCGTCATGTCACAA*	*TAGTAGGCCCCCTTCTGGTC*	NM_021022.3
ABCB4	*GAGCAAAGTCCAGGTCTGCG*	*TTGGTTGCTGATGCTGCCTA*	NM_008830.2
Cyp7a1	*GGGCAGGCTTGGGAATTTTG*	*AACGCTCAGCAGTCGTTACA*	NM_007824.2
Cyp27a1	*CGGGCAGAGAGTGAATCAGG*	*AGATCTGATGAAGGCGGCAG*	NM_024264.4
NPC1-L1	*GTGGAGTTCGTGTCCCACAT*	*TGAGCAAAGCCCAGGATGAG*	NM_207242.2
ACAT-2	*TTACCTCAGTCGCAGACAGG*	*TGAGCCCGCATTCATCTTGT*	NM_009338.3
β-ACTIN	*ACTCTTCCAGCCTTCCTTC*	*ATCTCCTTCTGCATCCTGTC*	

Table S1. Sequences of primers for real-time PCR. FABP4/ap2, adipocyte fatty acid binding protein 4; LPL, lipoprotein lipase; FAT/CD36, fatty acid translocase/ CD36 antigen; FSP27, fat specific protein 27; AdipoQ, adiponectin; ACC, acetyl-coenzyme A carboxylase; FASN, fatty acid synthase; SCD1, steroyl-coenzyme A desatures 1; DGAT-2, diacylglycerol O-acyltransferase 2; FATP-1, long-chain fatty acid transport protein 1; CPT-1a, carnitine-palmitoyl transferase1a; ACOX-1, acyl-Coenzyme A oxidase 1; mCAD, middle-chain acyl-CoA dehydrogenase; ApoB, apolipoprotein B; ApoC2, apolipoprotein C-II; ApoC3, apolipoprotein C-III; MTTP, microsomal triglyceride transfer protein; HL, hepatic lipase; ATGL, adipose triglyceride lipase; HSL, hormone sensitive lipase; SREBP-1, sterol regulatory element binding transcription factor 1; SREBP-2, sterol regulatory element binding transcription factor 1; PPARγ, peroxisome proliferator activated receptor gamma; PPARα, peroxisome proliferator activated receptor alpha; PPARβ/δ, peroxisome proliferator activated receptor delta; LXRα, liver X receptor alpha; LXRβ, liver X receptor beta; FXR, farnesoid X receptor; HMGCR, 3-hydroxy-3-methylglutaryl-Coenzyme A reductase; ApoA1, apolipoprotein A-I; ApoE, apolipoprotein E; SR-BI, scavenger receptor class B, member 1; LDLR, low density lipoprotein receptor; ABCA1, ATP-binding cassette, sub-family A , member 1; ABCG5, ATP-binding cassette, sub-family G, member 5; ABCG8, ATP-binding cassette, sub-family G , member 8; ABCB11, ATP-binding cassette, sub-family B (MDR/TAP), member 11; ABCB4, ATP-binding cassette, sub-family B (MDR/TAP), member 4; Cyp7a1, cytochrome P450, family 7, subfamily a, polypeptide 1; Cyp27a1, cytochrome P450, family 27, subfamily a, polypeptide 1; NPC1-L1, Niemann–Pick C1 Like 1; ACAT-2, acetyl-Coenzyme A acetyltransferase 2.
