# Supplementary material for: Effects and Potential Mechanisms of Pioglitazone on Lipid Metabolism in Obese Diabetic KKAy Mice
Source: PPAR Res. 2014 Mar 31;2014:538183. doi: 10.1155/2014/538183 (PMC3988943; doi:10.1155/2014/538183)
Supplement: Supplementary file 1 — Figure S1: Effects of pioglitazone on the levels of protein FASN, Acox1 and ACC in the liver of KKAy mice. Table S1: Sequences of primers for real-time PCR. [file 538183.f1.zip › mat.538183.v2.pdf]

Supporting information

Figure S1. Effects of pioglitazone on the levels of protein FASN, Acox1 and ACC in the liver of KKAy mice.

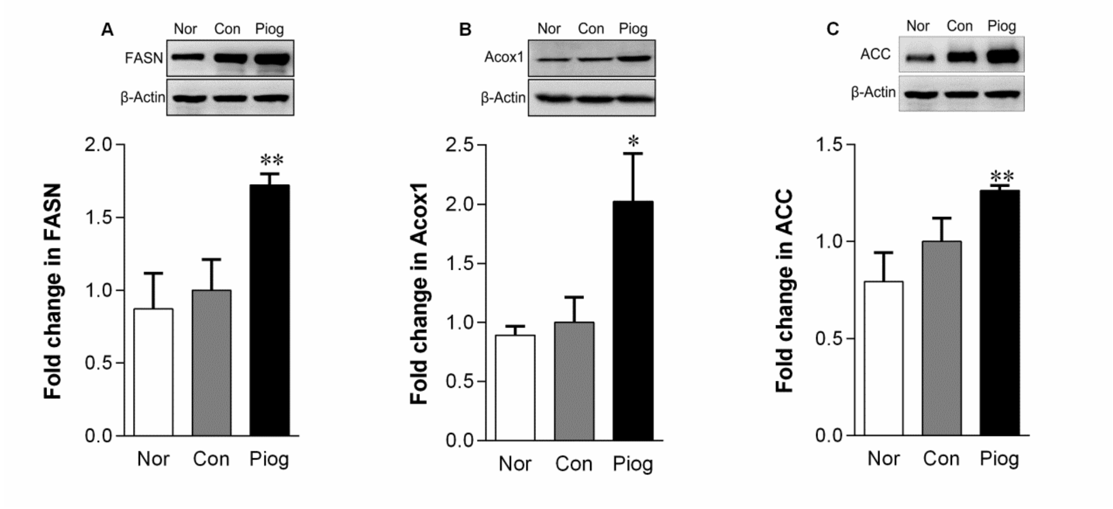

Figure S1. Effects of pioglitazone on the levels of protein FASN, Acox1 and ACC in the liver of KKAy mice. Representative Western blot for FASN (A), Acox1 (B), ACC (C) and β-actin. The bar graph represents statistical data from six individual mice per group. The bands were determined by densitometric analysis and expressed as fold change, after correction for β-actin levels, relative to the control mice. Nor, C57BL/6J mice; Con, vehicle-treated KKAy mice; Piog, pioglitazone-treated KKAy mice. Data are presented as mean ± S.E.M. *P<0.05, **P<0.01 vs. Con.

Table S1. Sequences of primers for real-time PCR.

**Gene**	**5'-Sense primer-3'**	**5'-Antisense primer-3'**	**Gene Bank Accession.Version**
FABP4/ap2	*TCACCATCCGGTCAGAGAGT*	*TTCATCGAATTCCACGCCCA*	NM_024406.2
LPL	*CGTAGTTCCAGCAGCAAAGC*	*GAAGGCGGTCAAACTCTGGA*	NM_008509.2
FAT/CD36	*CTCCTAGTAGGCGTGGGTCT*	*CACGGGGTCTCAACCATTCA*	NM_001159558.1
FSP27	*ATGGTGCCAGAGTGGTTAGC*	*AGAGTCCCAGGTGAGAGACC*	NM_178373.3
AdipoQ	*CCCAGTCATGCCGAAGATGA*	*CACAAGTTCCCTTGGGTGGA*	NM_009605.4
ACC	*GCTAAACCAGCACTCCCGAT*	*GTATCTGAGCTGACGGAGGC*	NM_133360.2
FASN	*CTTCTGGTGTGCAACTGTGC*	*AGGGCAGCTACCATGTTGTC*	NM_007988.3
SCD1	*GCGCTCTTTACCCTTTGCTG*	*GACTCTCGGGATGGGTGTTC*	NM_009127.4
DGAT-2	*ACTGGAACACGCCCAAGAAA*	*GTAGTCTCGGAAGTAGCGCC*	NM_026384.3
FATP-1	*CGCCGATGTGCTCTATGACT*	*ACACAGTCATCCCAGAAGCG*	NM_011977.3
CPT-1a	*GACCGCGCTCTTAGGACTAC*	*GAGCAGCAAGGAAGGACACT*	NM_013495.2
ACOX-1	*GGAGACAGGTTGTCATCGCT*	*TTCATGACGGAGACCAGTGC*	NM_015729.3
mCAD	*TCAAGATCGCAATGGGTGCT*	*GCTCCACTAGCAGCTTTCCA*	NM_007382.4

ApoB	*GTCCAGGTTGAATCACGGGT*	*AGGATCCTGCAAGGTCAAGC*	NM_009693.2
ApoC2	*GTCTCGGTTCTTCCTGGCTC*	*TCCTTGGCAGAGGTCCAGTA*	NM_009695.3
ApoC3	*GCTAAGTAGCGTGCAGGAGT*	*GCTCCAGTAGCCTTTCAGGG*	NM_023114.3
MTTP	*GTTTTTCCCGGTCAAGCGTT*	*TCTTTCAGTGGGGCGATCTT*	NM_001163457.1
HL	*GTCGCTGCTGGGAACAAAAG*	*GGATCAACTCGCCGATGTCT*	NM_008280.2
ATGL	*TGTGCAAACAGGGCTACAGAG*	*AAAGGGTTGGGTTGGTTCAG*	NM_001163689.1
HSL	*GTGAATGAGATGGCGAGGGT*	*GGAGTCGCGTTAGAGTCACC*	NM_010719.5
SREBP-1	*ACTTTTCCTTAACGTGGGCCT*	*CATCTCGGCCAGTGTCTGTT*	NM_011480.3
SREBP-2	*CATAGCACTGTCCCCGACTC*	*CTCCTTGGCTGCTGACTTGA*	NM_033218.1
PPARγ	*GTCACACTCTGACAGGAGCC*	*AGAACGTGACTTCTCAGCCC*	NM_001127330.1
PPARα	*CCGAACATTGGTGTTCGCAG*	*GATACGCCCAAATGCACCAC*	NM_011144.6
PPARβ	*ATGGGACTCACTCAGAGGCT*	*TGGCTGTTCCATGACTGACC*	NM_011145.3
LXRα	*AGAGTCTTGGGTCGCCAGTA*	*CTGGAGCCCTGGACATTACC*	NM_013839.4
LXRβ	*GCTAGCCATCATCTCGGTCC*	*TCTCAATGGTGGACGCCTTC*	NM_009473.2
FXR	*GGCTGCAAAGGTTTCTTCCG*	*ACATTCAGCCAACATCCCCA*	NM_001163700.1
HMGCR	*TGCCTGGATGGGAAGGAGTA*	*GCCTCGAGTCATCCCATCTG*	NM_008255.2
ApoA1	*GAACGAGTACCACACCAGGG*	*ATGGGCATCAGACTATGGCG*	NM_009692.3
ApoE	*CCTGAACCGCTTCTGGGATT*	*CCATCAGTGCCGTCAGTTCT*	NM_009696.3
SR-BI	*GTGCCCATCATCTGCCAACT*	*TAGGCCTGAATGGCCTCCTT*	NM_016741.2
LDLR	*GGGAACATTTCGGGGTCTGT*	*AGTCTTCTGCTGCAACTCCG*	NM_010700.3
ABCA1	*GCCTGGATCTACTCTGTCGC*	*GCCATTGTCCAGACCCATGA*	NM_013454.3
ABCG5	*CTATCAGCTTGTGGGTGCCA*	*GAGGACGTGTAGCACGTAGG*	NM_031884.1
ABCG8	*AAGACCCTTGGGCGTTACAG*	*CCACTGTGAAAGCCGATCCT*	NM_026180.2
ABCB11	*TCATCGCCGTCATGTCACAA*	*TAGTAGGCCCCCTTCTGGTC*	NM_021022.3
ABCB4	*GAGCAAAGTCCAGGTCTGCG*	*TTGGTTGCTGATGCTGCCTA*	NM_008830.2
Cyp7a1	*GGGCAGGCTTGGGAATTTTG*	*AACGCTCAGCAGTCGTTACA*	NM_007824.2
Cyp27a1	*CGGGCAGAGAGTGAATCAGG*	*AGATCTGATGAAGGCGGCAG*	NM_024264.4
NPC1-L1	*GTGGAGTTCGTGTCCCACAT*	*TGAGCAAAGCCCAGGATGAG*	NM_207242.2
ACAT-2	*TTACCTCAGTCGCAGACAGG*	*TGAGCCCGCATTCATCTTGT*	NM_009338.3
β-ACTIN	*ACTCTTCCAGCCTTCCTTC*	*ATCTCCTTCTGCATCCTGTC*	

Table S1. Sequences of primers for real-time PCR. FABP4/ap2, adipocyte fatty acid binding protein 4; LPL, lipoprotein lipase; FAT/CD36, fatty acid translocase/ CD36 antigen; FSP27, fat specific protein 27; AdipoQ, adiponectin; ACC, acetyl-coenzyme A carboxylase; FASN, fatty acid synthase; SCD1, steroyl-coenzyme A desatures 1; DGAT-2, diacylglycerol O-acyltransferase 2; FATP-1, long-chain fatty acid transport protein 1; CPT-1a, carnitine-palmitoyl transferase1a; ACOX-1, acyl-Coenzyme A oxidase 1; mCAD, middle-chain acyl-CoA dehydrogenase; ApoB, apolipoprotein B; ApoC2, apolipoprotein C-II; ApoC3, apolipoprotein C-III; MTTP, microsomal triglyceride transfer protein; HL, hepatic lipase; ATGL, adipose triglyceride lipase; HSL, hormone sensitive lipase; SREBP-1, sterol regulatory element binding transcription factor 1; SREBP-2, sterol regulatory element binding transcription factor 1; PPARγ, peroxisome proliferator activated receptor gamma; PPARα, peroxisome proliferator activated receptor alpha; PPARβ/δ, peroxisome

proliferator activated receptor delta; LXRα, liver X receptor alpha; LXRβ, liver X receptor beta; FXR, farnesoid X receptor; HMGCR, 3-hydroxy-3-methylglutaryl-Coenzyme A reductase; ApoA1, apolipoprotein A-I; ApoE, apolipoprotein E; SR-BI, scavenger receptor class B, member 1; LDLR, low density lipoprotein receptor; ABCA1, ATP-binding cassette, sub-family A , member 1; ABCG5, ATP-binding cassette, sub-family G, member 5; ABCG8, ATP-binding cassette, sub-family G , member 8; ABCB11, ATP-binding cassette, sub-family B (MDR/TAP), member 11; ABCB4, ATP-binding cassette, sub-family B (MDR/TAP), member 4; Cyp7a1, cytochrome P450, family 7, subfamily a, polypeptide 1; Cyp27a1, cytochrome P450, family 27, subfamily a, polypeptide 1; NPC1-L1, Niemann–Pick C1 Like 1; ACAT-2, acetyl-Coenzyme A acetyltransferase 2.
